# Upregulation of Cardiomyocyte Ribonucleotide Reductase Increases Intracellular 2 deoxy-ATP, Contractility, and Relaxation

**DOI:** 10.1016/j.yjmcc.2011.08.026

**Published:** 2011-09-03

**Authors:** F. Steven Korte, Jin Dai, Kate Buckley, Erik R. Feest, Nancy Adamek, Michael A. Geeves, Charles E. Murry, Michael Regnier

**Affiliations:** 1 Department of Bioengineering, University of Washington, Seattle WA 98195 USA; 2 Department of Pathology, University of Washington, Seattle WA 98195 USA; 3 Department of Medicine/Cardiology, University of Washington, Seattle WA 98195 USA; 4 Department of Biosciences, University of Kent, Canterbury, Kent CT2 7NJ UK; 5 Center for Cardiovascular Biology, Institute for Stem Cell and Regenerative Medicine, University of Washington, Seattle WA 98109 USA

**Keywords:** Cardiomyocyte, Contractility, Ribonucleotide Reductase

## Abstract

We have previously demonstrated that substitution of ATP with 2 deoxy-ATP (dATP) increased the magnitude and rate of force production at all levels of Ca^2+^-mediated activation in demembranated cardiac muscle. In the current study we hypothesized that cellular [dATP] could be increased by viral-mediated over expression of the ribonucleotide reductase (Rrm1 and Rrm2) complex, which would increase contractility of adult rat cardiomyocytes. Cell length and ratiometric (fura2) Ca^2+^ fluorescence were monitored by video microscopy. At 0.5 Hz stimulation, the extent of shortening was increased ~40% and maximal rate of shortening was increased ~80% in cardiomyocytes overexpressing Rrm1+Rrm2 as compared to non-transduced cardiomyocytes. The maximal rate of relaxation was also increased ~150% with Rrm1+Rrm2 over expression, resulting in decreased time to 50% relaxation over non-transduced cardiomyocytes. These differences were even more dramatic when compared to cardiomyocytes expressing GFP-only. Interestingly, Rrm1+Rrm2 over expression had no effect on minimal or maximal intracellular [Ca^2+^] (Fura2 fluorescence), indicating increased contractility is primarily due to increased myofilament activity without altering Ca^2+^ release from the sarcoplasmic reticulum. Additionally, functional potentiation was maintained with Rrm1+Rrm2 over expression as stimulation frequency was increased (1 Hz and 2 Hz). HPLC analysis indicated cellular [dATP] was increased by approximately 10-fold following transduction, becoming ~1.5% of the adenine nucleotide pool. Furthermore, 2% dATP was sufficient to significantly increase crossbridge binding and contractile force during sub-maximal Ca^2+^ activation in demembranated cardiac muscle. These experiments demonstrate the feasibility of directly targeting the actin-myosin chemomechanical crossbridge cycle to enhance cardiac contractility and relaxation without affecting minimal or maximal Ca^2+^.

## 1. INTRODUCTION

Systolic and/or diastolic cardiac function is compromised in a number of cardiovascular diseases including myocardial infarction, ischemia/reperfusion injury, diabetes, high blood pressure and hypertrophic and dilated cardiomyopathy. These pathophysiological conditions often involve alterations in the Ca^2+^ cycle[1], β-adrenergic responsiveness[2], and/or the contractile apparatus of cardiomyocytes[3, 4]. To date, therapeutic efforts have focused primarily on approaches that increase [Ca^2+^]_i_ that can be pro-arrhythmogenic and may impair ventricular filling by slowing diastolic relaxation[5]. Other approaches involving adrenergic agents can have undesirable long-term consequences, including significant side-effects due to drug actions in non-target areas, pro-arrythmogenic triggered activity, and potential for accelerated progression into heart failure[2]. Thus, new approaches to combat cardiac dysfunction are desirable.

We have previously shown that replacing ATP with 2 deoxy-ATP (dATP) as the substrate for contraction of demembranated cardiac muscle increased isometric force and the rate of force development and shortening at all levels of Ca^2+^ activation, including saturating [Ca^2+^] (pCa 4.0) [6–9]. The presence of dATP results in enhanced myosin binding to actin and an increase in the rates of Pi and dADP release and myosin detachments. As such, contractile properties can be improved by >50% over the range of [Ca^2+^]_i_ seen *in vivo.* Thus, replacement of ATP with dATP offers the potential to improve contraction independent of changes in [Ca^2+^]_i_ or adrenergic signaling.

To date, the effect of dATP has only been studied in demembranated cardiac tissue and with isolated contractile proteins. As such, its potential to improve intact cardiomyocyte contraction or cardiac function *in situ* is unknown. Cellular production of dATP occurs in the cytoplasm of mammalian cells by ribonucleotide reductase (Rrm), which removes a hydroxyl moiety from the 2-position on the ribose ring of ADP to produce dADP. dADP is then rapidly converted to dATP. Rrm consists of two subunit proteins, a catalytic activator (Rrm1) and free radical containing (Rrm2) subunit and is regulated by nucleoside triphosphate allosteric effectors[10]. While the details of regulating cellular RR content, enzymatic activity and cellular concentration [dATP] are unclear, it is known that both subunits are necessary for activity.[11]

In the current study, we produced adenoviral vectors expressing cytomegalovirus (CMV) promoter driven Rrm1 or Rrm2, each along with green fluorescent protein (GFP) as a transduction reporter. Cultured adult rat cardiomyocytes were transduced with these vectors, and the rate and extent of myocyte contraction and relaxation and Ca^2+^ transient rise and decay (Fura2 fluorescence) were monitored by video microscopy following a 48 hour viral incubation period. Here we show that these treatments significantly increased cellular [dATP], rate and extent of shortening, and rate of relaxation, with minimal effects on Ca^2+^ transients, at 0.5 Hz, 1 Hz and 2 Hz stimulation. Additionally, the [dATP] found in transduced cells (1–2% of adenine nucleotide content) was sufficient to increase sub-maximal Ca^2+^ activated force in skinned cardiac trabeculae. These experiments suggest that increases in cardiac intracellular Rrm and/or the dATP pool can significantly alter the actin-myosin crossbridge cycle to enhance cardiac contractility without impairing diastolic function or cardiomyocyte Ca^2+^ handling.

## 2. METHODS

Greater details of plasmid design and vector production, cell culture, contractile assessment, nucleotide binding affinity, and western blot analysis are provided in online supporting information.

### 2.1 Animal and Tissue Handling

These studies were approved by the University of Washington (UW) Animal Care Committee and conducted in accordance with federal guidelines. Animals were cared for in accordance with US NIH Policy on Humane Care and Use of Laboratory Animals in the Department of Comparative Medicine at UW. Adult rat (Fischer 344) cardiomyocytes (ARCs) were isolated from heart using aortic retrograde perfusion for enzymatic (collagenase/protease) dispersion of cells[12]. Neonatal Rat Cardiomyocytes (NRCs) were isolated by enzymatic dispersion from 1–3-day old newborn Fischer 344 rats as previously described[13]. Rat cardiac trabeculae were dissected from the right ventricle of male Sprague-Dawley rats, chemically demembranated, and prepared for mechanical measurements as previously described [13]. Trabeculae averaged 1.3 ± 0.2 mm in length by 170 ± 30 μm in width.

### 2.2 Plasmid design and virus production

HEK293 cells were used to generate adenoviral vectors[14] expressing Rrm1 or Rrm2 from the CMV promotor. Both vectors contained a second expression cassette for green fluorescent protein (GFP) as a transduction reporter protein, and we also expressed a vector for GFP-only. Virus was introduced to cardiomyocytes at ~250 particles per cell.

### 2.3 Nucleotide Binding Affinity

Rapid kinetic measurements of nucleotide binding and actin-myosin dissociation were taken at 10°C and 20°C (Hi-Tech Scientific SF-61 DX2 stopped-flow system) as previously described[15] using pyrene labeled actin and myosin S1. Myosin was purified from mouse hearts, rabbit soleus, and rabbit bulk fast muscle as previously described[16, 17]. Actin was purified from rabbit skeletal muscle[18]. The stopped-flow transients were fitted to one or two exponentials by non-linear least squares curve fitting using the Kinetic Studio software (TgK Scientific). All experiments were carried out in 20 mM Cacodylate buffer, pH 7.0 containing 100 mM KCl, and 5 mM MgCl_2_. The rate constant for ATP-induced actin-S1 dissociation (k_obs_) was determined from equation 1 based on SI Scheme 1:

Equation 1 $${\text{k}}_{\text{obs}}={\text{k}}_{+2}{\text{K}}_{1}\frac{\left[\text{ATP}\right]}{1+{\text{K}}_{1}[\text{ATP}]}$$

### 2.4 Contractile Assessments

In modified Tyrodes buffer at ambient temperature (22–24°C) and at 37°C, cell shortening and relaxation of arbitrarily selected stimulated cardiomyocytes was recorded using IonOptix system video microscopy. (IonOptix, Milton, MA, USA). Calcium transients induced by electrical stimulation were measured in Fura2 loaded cells using IonOptix equipment as described[19]. Fura2 fluorescence was measured using an IonOptix spectrophotometer (Stepper Switch) attached to a fluorescence microscope. Emitted Fura2 fluorescence was collected by the 40X objective, passed through a 510nm filter and detected by a photomultiplier tube. For demembranated trabeculae, steady-state force and high frequency sinusoidal stiffness (to determine crossbridge binding) were measured in a custom built mechanical apparatus at 15° and 22° C during sub-maximal (pica 5.6) and maximal (pica 4.0) Ca^2+^ activation as previously described [20]. Experimental physiological Ca^2+^ solutions were calculated as previously described for trabeculae mechanics [21].

### 2.5 Data Processing and Statistical Analysis

Maximal cardiomyocyte shortening and relengthening and calcium transient rise and decay were calculated offline using IonOptix software to determine the maximum of the first derivative of these transients. Times to peak shortening and 50% and 90% return to baseline were also calculated offline. Statistical differences were determined by ANOVA, with Student-Newman-Keuls as a *post-hoc* pairwise test (SigmaPlot 11). Trabeculae were compared using paired t-tests. Differences at p-value < 0.05 were considered statistically significant. Data is displayed as mean ± s.e.m.

## 3. RESULTS

Transudation with recombinant adenovirus containing appropriate coda constructs driven by the CMV promoter was used to induce over expression of muscle rib nucleotide reeducates 1 (Rrm1) and 2 (Rrm2) in cultured adult and neonatal rat cardiomyocytes. Each adenovirus also contained a second expression cassette for green fluorescent protein (GFP), which was used as a reporter protein identifying successful transduction. Cardiomyocytes were infected with adenovirus containing genes for [Rrm1 + GFP and Rrm2 + GFP] or [GFP] for 2 days. Successful gene transfer, grossly indicated by green fluorescence with microscopy, indicated nearly 100% transduction efficiency (Supplemental Figure 1). This is consistent with previous studies using cardiomyocytes[22]. Cell survival over this period was similar for all groups, including non-transduced control cells, suggesting these viral vectors did not compromise cardiomyocyte viability. Cardiomyocyte numbers and sarcomere lengths are summarized in Table 1. There was no difference in resting sarcomere length between groups, indicating that over expression of Rrm1+Rrm2 (or GFP) did not increase calcium independent activation.

### 3.1 Contractile Analysis of Cultured Cardiomyocytes

**Figure 1:**
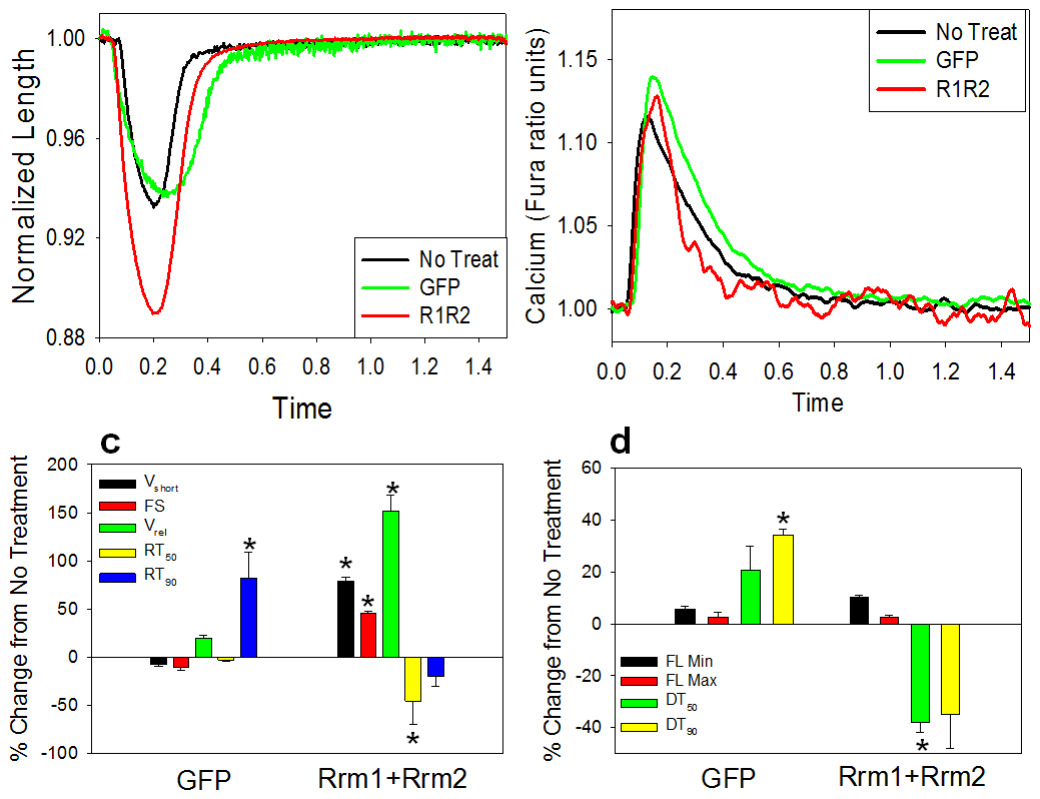
Representative traces and data summary Representative cell length traces **(a)** and Ca^2+^ transients **(b**, Fura-2 fluorescence) of non-transducer (black), GFP-only (green), and Rrm1+Rrm2+GFP (red) transducer cardiomyocytes. Percentage change in contractile **(c)** and Ca^2+^ transient **(d)** properties of GFP-only and Rrm1+Rrm2+GFP transducer myocytes, stimulated at 0.5 Hz, as compared to non-transducer myocytes. V_short_ = velocity of shortening; FS = fractional shortening; V_rel_ = maximal relaxation velocity; RT_50,90_ = time to 50% and 90% relaxation, respectively; FL = fluorescence; DT_50,90_ = time to 50% and 90% Ca^2+^ decay, respectively *p<0.05 as compared to Non-Transducer.

The effects of Rrm1+Rrm2 over expression on extent and rate of stimulated shortening-relengthening of adult rat cardiomyocytes were determined using video length-detection (IonOptix). Figure 1a shows representative shortening traces, and Figure 1b shows representative Ca^2+^ transients (Fura2 fluorescence), for non-transduced (black), GFP-only (green), and Rrm1+Rrm2 (red) transduced cardiomyocytes. The data for all measurements at 0.5 Hz is summarized in Table 2. Cardiomyocytes transducer with Rrm1+Rrm2 (+GFP) had a significantly greater magnitude and rate of shortening vs. non-transducer cardiomyocytes and GFP-only transducer controls. This is illustrated in Figure 1c, which shows % differences in rate and extent of shortening, relaxation rate, and time to 50% and 90% relaxation. While GFP has been reported to have a deleterious[23] or no effect[19, 24] on contractility, it did not appear to act as a contractile inhibitor in this study. However, GFP did slow the 90% relaxation time, which was accompanied by a slower time to 50% and 90% decay of the Ca^2+^ transient (Figure 1c, Table 2). Regardless, Rrm1+Rrm2 over expression increased the rate of relaxation and decreased the time to 50% relaxation, and this effect may be somewhat underestimated due to the presence of GFP. Figure 1d illustrates the % difference in Ca^2+^ transient properties, including minimal and maximal Ca^2+^, and the time to 50% and 90% Ca^2+^ decay. There was no significant effect from either GFP or Rrm1+Rrm2 + GFP on minimal and maximal Ca^2+^, indicating that enhanced contractility with Rrm1+Rrm2 was primarily due to increased myofilament responsiveness to activating Ca^2+^. Interestingly, Rrm1+Rrm2 over expression did speed Ca^2+^ re-sequestration, as indicated by a reduction the time to 50% and 90% decay. This could be due to increased SERCA activity and, in part, explain the increased maximal rate of cardiomyocyte relaxation at 0.5 Hz (Table 2) stimulation. Faster relaxation in Rrm1+Rrm2 overexpressing cardiomyocytes could also be due to faster crossbridge cycling, that leads to shortening induced thin filament inactivation and Ca^2+^ release from troponin C.

**Figure 2:**
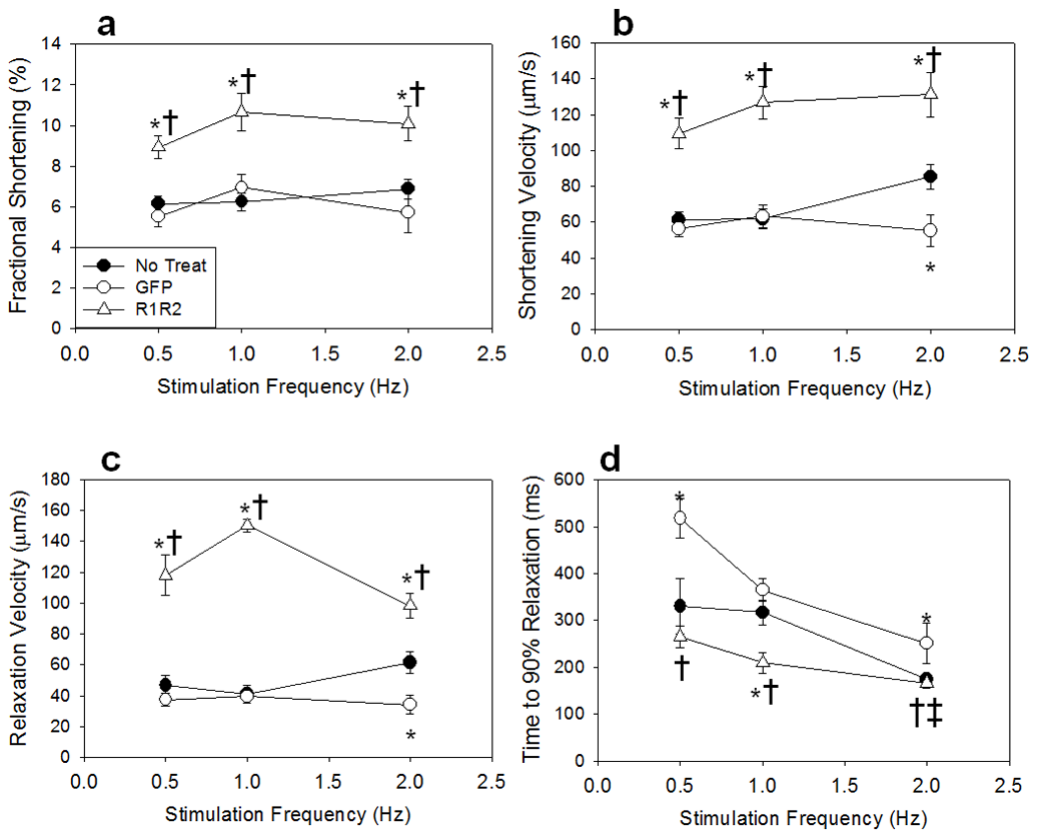
Effect of stimulation frequency on contractile properties Rrm1+Rrm2 transducer myocytes (open triangles) respond similarly to stimulation frequency as GFP-only transduce open circles) and non-transducer myocytes (closed circles) but show elevated fractional shortening **(a)** and shortening velocity **(b)** at all frequencies. Relaxation velocity **(c)** and time to 90% relaxation **(d)** are also similar between groups, with time to relaxation shortening as stimulation frequency increases. * = p<0.05 as compared to Non-Transducer, † = p<0.05 as compared to GFP, ‡ = p<0.05 as compared to 0.5 Hz for all groups.

**Figure 3:**
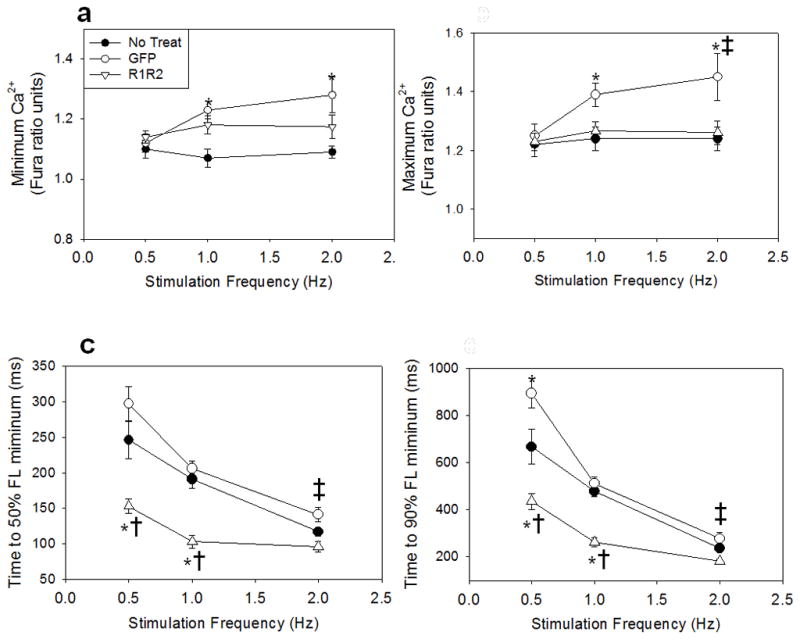
Effect of stimulation frequency on Ca^2+^ handling properties Rrm1+Rrm2 transducer myocytes (open triangles) respond similarly to stimulation frequency as non-transducer myocytes (closed circles) in minimal **(a)** and maximal **(b)** fluorescence, while GFP-only transducer myocytes (closed circles) showed a greater increase in both as frequency increased. As with cardiomyocyte relaxation, Ca^2+^ transient decay time (DT) to 50% **(c)** and 90% **(d)** is shortened with increased stimulation frequency, but both are dramatically shortened in R1R2 transducer cardiomyocytes. * = p<0.05 as compared to Non-Transducer, † = p<0.05 as compared to GFP, ‡ = p<0.05 as compared to 0.5 Hz for all groups.

It is important to determine whether Rrm1+Rrm2 over expression affects normal cellular response to increased stimulation frequency, as changes in heart rate are a normal physiological adaptation to systemic demand. Figure 2 summarizes the effect of increased stimulation frequency (0.5 to 1 to 2 Hz) on fractional shortening (2a), shortening velocity (2b), relaxation velocity (2c), and time to 90% relaxation (2c). The contractile response to stimulation frequency was similar between groups, and Rrm1+Rrm2 transducer cardiomyocytes maintained functional potentiation at all frequencies. Importantly, increased pacing frequency is associated with a positive lusitropic effect, shortening the time to 90% relaxation in all groups. There was little difference in non-transducer myocytes vs. GFP-only transducer myocytes, except that time to 90% relaxation is longer at 0.5 Hz in GFP-only myocytes. The effect of stimulation frequency on Ca^2+^ transients was also assessed, and is summarized in Figure 3 for minimal Ca^2+^ (3a), maximal Ca^2+^ (3b) and time to 50% (3c) and 90% (3d) Ca^2+^ decay (DT_50_, DT_90_). As with contraction, there was no difference in Ca^2+^ transient behavior with increased stimulation frequency between non-transducer and Rrm1+Rrm2 transducer myocytes. GFP-only transducer cardiomyocytes had a slight increase in minimal Ca^2+^ at 2 Hz, and an increase in maximal Ca^2+^ at 1 Hz and 2 Hz, as compared to non-transducer myocytes, but the times to 50% and 90% decay were similar. As at 0.5 Hz, the times to 50% and 90% decay were decreased (faster decay) in Rrm1+Rrm2 transducer myocytes at both 1 and 2 Hz. Most importantly, although higher stimulating frequencies increased relaxation parameters in all groups, the relative increase in relaxation kinetics was maintained with Rrm1+Rrm2 over expression, such that relaxation was improved, not impaired. Results for 1 Hz and 2 Hz stimulation are summarized in Supplemental Table 1 and 2, respectively.

We chose to perform these experiments at room temperature (22–24° C) to compare with the predominant number of reports for cultured cardiomyocytes in the literature [25–29]. However, a subset of measurements was made at 37°C to determine if the effects persist at physiological temperature. At 37°C (Supplemental Table 3), shortening and Ca^2+^ transients were faster than at 22–24°C, but were similarly increased in cardiomyocytes transducer with Rrm1+Rrm2 vs. GFP-only transducer and non-transducer cells. Similarly, the rates of Ca^2+^ release and re-uptake were also increased at 37°C vs. room temperature, but with Rrm1+Rrm2 over expression resulting in faster Ca^2+^ transient decay as was observed at ambient temperature.

**Figure 4:**
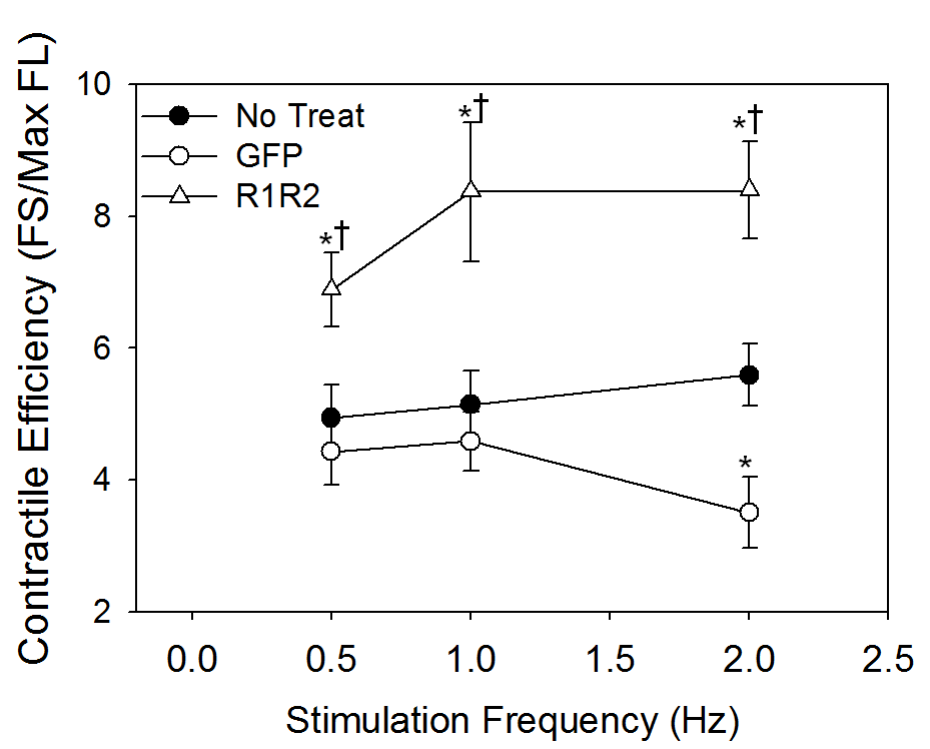
Contractile Responsiveness Contractile response as assessed as fractional shortening divided by maximal fura fluorescence (peak Ca^2+^) indicates Rrm1+Rrm2 transducer cardiomyocytes (open triangles) are significantly more responsive to Ca^2+^ at all stimulation frequencies, while GFP-only transducer cardiomyocytes (open circles) are less responsive to Ca^2+^ only at 2Hz stimulation frequency as compared to non-transducer cardiomyocytes (closed circles). * = p<0.05 as compared to Non-transducer, † = p<0.05 as compared to GFP.

Since there was little difference between groups in minimal and maximal Ca^2+^, changes in contractility can best be explained by a change in myofilament responsiveness to activating Ca^2+^. This is illustrated in Figure 4 as contractile response, defined here as cardiomyocyte fractional shortening divided by maximal fura2 fluorescence (peak Ca^2+^). Cardiomyocytes expressing Rrm1+Rrm2 had significantly higher contractile response than non-transducer or GFP transducer cardiomyocytes at all stimulation frequencies. There was no difference in contractile response between GFP only or non-transducer myocytes except at 2 Hz, which can be primarily be attributed to increased maximal Ca^2+^ in GFP only myocytes with no increase in fractional shortening, reducing response.

### 3.2 Protein and Nucleotide Analysis

**Figure 5:**
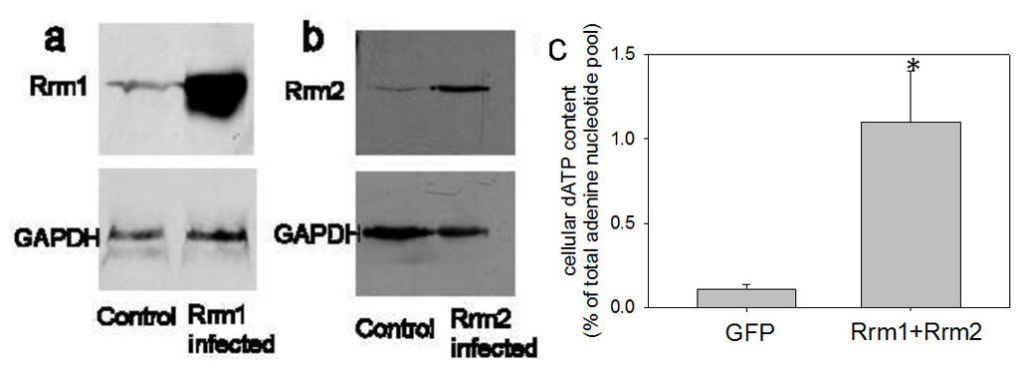
Increased Rrm and dATP **(a)** Western blot of Rrm1 transducer neonatal rat cardiomyocytes probed with anti-Rrm1 antibody indicates a >24-fold increase in Rrm1. **(b)** Western blot of Rrm2 transducer neonatal rat cardiomyocytes probed with anti-Rrm2 antibody indicates a > 46-fold increase in Rrm2. **(c)** Rrm1+Rrm2 over expression significantly increased intracellular [dATP] by >10-fold in neonatal rat cardiomyocytes as assessed by HPLC analysis. * = p<0.05 as compared to GFP transducer cardiomyocytes.

To verify increased Rrm mRNA, Rrm protein, and dATP production in Rrm1+Rrm2 transducer cells, neonatal rat cardiomyocytes were collected and processed for RT-PCR, western blotting and HPLC analysis of intracellular [ATP] and [dATP]. Neonatal cardiomyocytes were used to achieve high enough cell density for accurate nucleotide content analysis, as intracellular [dATP] is known to be in the pM range. Although there are structural differences between neonatal and adult cardiomyocytes, it is important to note that Rrm1+Rrm2 over-expression increased contractility to a similar extent in both cell types (Supplemental Figure 2). Interestingly, as neonatal cardiomyocytes have been used to study the effects of cellular engraftment following myocardial infarction[13], improved contractility in these cells may be another mechanism to improve cardiac function following an infarct. Rrm1 and Rrm2 mRNA was significantly increased following adenoviral tranduction (Supplemental Figure 3). Concomitant with this, Figure 5a and 5b illustrate that Rrm1 and Rrm2 transducer cardiomyocytes had greater than 24-fold and 46-fold increased Rrm1 and Rrm2 protein content, respectively. GAPDH was used as a loading control. Figure 5c illustrates that Rrm1+Rrm2 transducer cardiomyocytes had ~10-fold increased cellular [dATP] as compared to GFP transducer cardiomyocytes (an increase to 0.35 nmol/mg protein). While this is robust, since [dATP] normally comprises less than 0.2% of total adenine triphosphate nucleotide, this increase in [dATP] represents only ~1.5% of the total adenine nucleotide pool. This suggests that only a small amount of dATP is required to significantly increase cardiomyocyte contractility.

**Figure 6:**
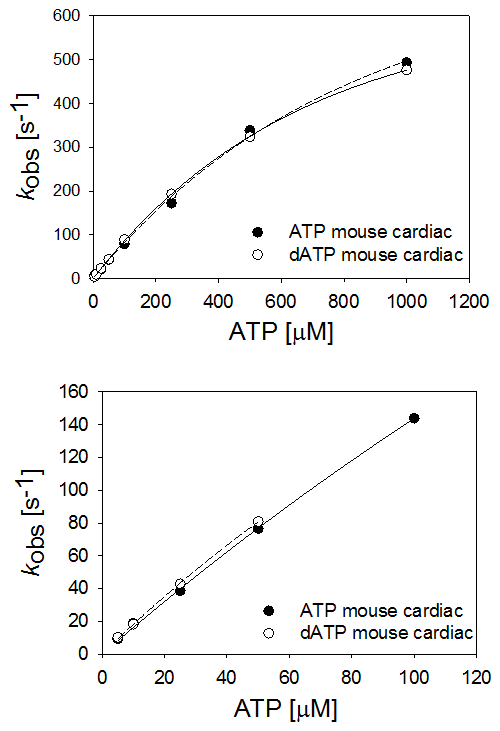
Nucleotide binding and actin-myosin dissociation Rapid kinetic measurements of nucleotide binding and actin-myosin dissociation of mouse cardiac myosin taken at 10°C (top) and 20°C (bottom). There was no difference in k_obs_ between ATP and dATP at any [NTP] at either temperature.

To determine how the relatively small increase in cellular [dATP] might influence crossbridge binding and contraction, we compared the rates of nucleotide binding + acto-myosin S1 dissociation (k_obs_) for ATP vs. dATP. Figure 6 shows the effect of increasing [ATP] and [dATP] on k_obs_ at 10°C and 20°C for mouse cardiac (alpha) myosin. There was no difference in k_obs_ between ATP and dATP at any [NTP] at either temperature. This was also true for fast and slow skeletal S1 myosin (Supplemental Figure 4). This data indicates NTP binding to S1, and subsequent S1 dissociation from actin, is not different for dATP vs. ATP. Thus, it appears that enhanced contractility of R1R2 overexpressing cardiomyocytes is not likely due to a greater myosin affinity for dATP.

### 3.3 Crossbridge Binding and Force in Demembranated Trabeculae

**Figure 7:**
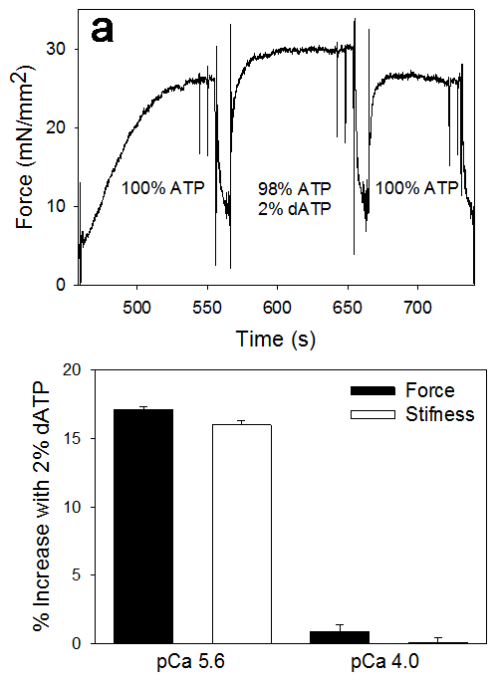
Isometric force increases with 2% dATP Isometric force development of demembranated cardiac trabeculae activated with 2% dATP, 98% ATP vs. 100% ATP (5 mM [NTP] total). **(a)** Force trace with pica 5.6 activation. **(b)** Summary of % increase in force with 2% dATP in activation solutions for sub-maximal (pica 5.6), but not maximal (pica 4.0) Ca^2+^ activation.

To determine if small amounts of dATP increase force production, we activated contraction of demembranated rat cardiac trabeculae at pica 5.6 (sub-maximal) and pica 4.0 (maximal) in solutions containing 5 mM NTP, composed of either 100% ATP or 2% dATP, 98% ATP. The sub-maximal Ca^2+^ activation approximates the force levels attained during twitch activity, and was 30 ± 7% of maximal force (88.4 ± 1.9 mN/mm^2^). The example force trace in Figure 7A demonstrates that moving a trabeculae from 100% ATP solution to the 2% dATP, 98% ATP solution resulted in a significant increase in force, which was reversible upon tansfer back to the 100% ATP solution. Figure 7B summarizes this increase for all trabeculae activated and shows that 2% dATP, 98% ATP increased force 17.1 ± 0.02% (p<0.05) at pica 5.6 but not during maximal Ca^2+^ (pica 4.0) activation. Similarly, crossbridge binding, assessed by high frequency sinusoidal stiffness measurements, increased 16.0 ± 0.03% (p<0.05), which indicates that the increased force with 2% dATP, 98% ATP is due to increased crossbridge binding. Thus the data demonstrate that a relatively small increase in cellular [dATP] (1–2% of adenine nucleotide) is sufficient to significantly increase the contractile strength of intact cardiomyocytes by increasing the number of strong crossbridges.

## 4. DISCUSSION

The main objective of this study was to determine if over expression of rib nucleotide reeducates (Rrm1+Rrm2) increases cellular [dATP] and, in turn, increases contractility in intact cardiomyocytes without adversely affecting cardiomyocyte relaxation. Over expression of Rrm1+Rrm2 resulted in increased cellular [dATP] to ~1.5% of the total adenine nucleotide pool, and this dramatically increased the extent and rate of myocyte shortening and rate of myocyte relaxation, while having no apparent effect Ca^2+^ transient properties.

Previous experiments in our laboratory using skinned cardiac trabeculae showed dATP increased isometric force and the rate of force development and shortening at all levels of Ca^2+^ activation, including saturating [Ca^2+^] (pica 4.0), but these studies were performed with 100% replacement of 5 mM ATP with 5 mM dATP in bathing solutions[6–8]. For our current study in intact cardiomyocytes we did not expect over expression of Rrm1+Rrm2 to result in high (mM) levels of dATP. Others have shown that as little as 10% replacement of ATP with dATP is sufficient to see a gain of force in demembranated porcine trabeculae (15°C)[30] and replacement of ~30% increased contractility in intact embryonic chick cardiomyocytes[31]. In our studies the observed large increases in contractility of adult rat cardiomyocytes occurred with a relatively small increase in cellular [dATP] resulting from over expression of Rrm1+Rrm2, and a similar amount (2% dATP) significantly increased sub-maximal force in dememembranated trabeculae. It is possible there was a small population of contaminating cells (e.g., fibroblasts) that were either not as easily transducer or overexpressed less Rrm1+Rrm2, which would lead to underestimation of cardiomyocyte [dATP] from the HPLC analysis. However, considering the relative scarcity of non-cardiomyocyte cells in our culture, this confounding effect should be minimal. Our force measurements with demembranated trabeculae suggest this relatively small concentration of cellular [dATP] is sufficient to result in the increased contractility seen in intact cardiomyocytes. This may be advantagous in that large increases in [dATP] are not required to achieve contractile potentiation, thus reducing the potential for negative side effects[31, 32].

It is interesting to speculate on how a relatively small increase in cellular dATP can improve cardiomyocyte function. Contractile response estimations (Figure 4) indicate increased contractility in Rrm1+Rrm2 transducer myocytes is primarily myofilament based, thus dATP likely has its effect primarily by improving myosin binding (to actin) and crossbridge cycling. The increase in sub-maximal steady-state force and stiffness seen in demembranated trabeculae activated with 2% dATP, 98% ATP supports this idea. This is similar to experiments where faster (alpha) myosin has been expressed in cardiomyocytes that normally express slower (beta) myosin, resulting in functional potentiation with no effect on Ca^2+^ transient amplitude [24]. One possibility we examined was that dATP affinity for cardiac myosin is much greater than ATP affinity for myosin, such that the increased level in cells (with Rrm1+Rrm2 over expression) was utilized almost specifically by myosin. We have previously shown that ATP and dATP have similar binding affinity to skeletal myosin and actomyosin and a similar γphosphate cleavage equilibrium by myosin [33]. Here we report that NTP binding and subsequent dissociation of cardiac α-myosin from actin does not differ for dATP vs. ATP, as assessed by k_obs_ (Figure 6). This suggests dATP has a similar binding affinity for cardiac myosin as ATP (Supplemental Scheme 1). For skeletal myosin we have also shown post-hydrolysis crossbridge binding and the rate of crossbridge detachment is increased with dATP[33]. This can explain an increase in the Ca^2+^ sensitivity of tension development, and a faster rate of tension development and shortening velocity in skinned skeletal muscle[33–35]. While we have not performed a detailed chemo-mechanical analysis with dATP in cardiac muscle, we have shown that it increases maximal crossbridge binding (as indicated from stiffness measurements) and isometric force by >40%, in addition to increasing *k_tr_* and unloaded shortening velocity[6]. We have also shown that dATP significantly increases isometric force and *k_tr_* in cardiac muscle at all levels of Ca^2+^, whether the demembranated cardiac muscle was expressing primarily α-or β-myosin heavy chain[8]. This is important because, unlike skeletal muscle, the intracellular [Ca^2+^] during a cardiac muscle twitch only reaches a level that is approximately within the half-maximally activating range. Additionally, cooperative thin filament activation in cardiac muscle is strongly influenced by strong binding crossbridges[7]. Based on our results that 2% dATP, 98% ATP significantly enhanced crossbridge binding and force in demembranated trabeculae, we propose that dATP results in the formation of a few additional strong binding crossbridges early on during the twitch, which provides a positive feedback amplification of thin filament activation. This results in greater total crossbridge binding, including crossbridges using ATP, during the cardiomyocyte twitch. Therefore, for our current experiments with cultured cardiomyocytes, it may be that a small increase in initial binding of myosin S1 heads was enough to cooperatively increase thin filament activation, resulting in the increased magnitude and rate of shortening. Future studies will be required to determine how R1R2 over expression and resulting increases in cellular [dATP] affect cardiac function *in situ*, but previous experiments have demonstrated that even demembranated cardiomyocyte function translates to cardiac organ function[36]. Interestingly, a recent study investigating a small molecule myosin activator (omecamtiv mecarbil) demonstrated functional potentiation at the cardiomyocyte level, via increased crossbridge binding, that was very similar to results in the present study[37]. However, this molecule appeared to slow the times to peak shortening and 50% and 90% relaxation, which would likely increase time spent in systole while decreasing time spent in diastole. In contrast, Rrm1+Rrm2 over-expression increased fractional shortening without increasing the time to peak shortening, and shortened the times to 50% and 90% relaxation (Figure 1a, Table 1, Supplemental Tables 1–4), which would allow more time for ventricular filling.

There was no adverse effect on relaxation with over expression of Rrm1+Rrm2 (and the subsequent increase in [dATP]), in fact myocyte relaxation was enhanced. It is possible that this resulted, at least in part, from a faster decay of the Ca^2+^ transient. dATP could be used by other ATPases (besides myosin) such as the sarcoplasmic Ca^2+^ ATPase (SERCA), the plasma membrane Ca^2+^ ATPase (PMCA), and may also indirectly effect activity of the sodium/calcium exchanger (NCX)[38]. An increase in SERCA activity could explain the increased decay rate of the Ca^2+^ transient, especially at 0.5 and 1.0 Hz stimulation. However, increased SERCA activity is known to increase SR Ca^2+^ stores[39], making more Ca^2+^ available for release during activation, which was not observed in Rrm1+Rrm2 transducer cardiomyocytes (Figure 1d). Furthermore, increased PMCA and NCX activity should result in a Ca^2+^ transient decay over time by extruding Ca^2+^ out of the cell. Because ~95% of activating Ca^2+^ is released from the SR in rat cardiomyocytes[40, 41], Ca^2+^ extrusion from the cell would lead to progressively decreased Ca^2+^ transient amplitudes and contraction, which was not observed over the duration of these experiments. However, the specific mechanism behind increased Ca^2+^ transient decay rate warrants future investigation.

Since dATP increases the rate of crossbridge detachment[6, 34] this may also explain a faster rate of relaxation in the current experiments with cultured cardiomyocytes. Although specific mechanisms that govern relaxation in intact cardiac muscle are not known, early phase relaxation in cardiac and skeletal myofibrils has been shown to be governed by the rate of crossbridge dissociation[42–45]. This would also be consistent with our finding that cardiomyocyte contractility was increased with Rrm1+Rrm2, because shortening rate in unloaded cells (as in culture) is primarily determined by crossbridge detachment rates[46, 47], although cultured cardiomyocytes still contract against a small internal load. It is also possible that increased crossbridge detachment rate with dATP accelerates cooperative thin filament inactivation, by more rapidly decreasing the bound crossbridge population as thin filament Ca^2+^ binding decreases during relaxation. A more rapid decrease in bound crossbridge population could also increase the rate of Ca^2+^ dissociation from troponin, as demonstrated by Tikunova *et al*. (2010) [48], further accelerating relaxation.

In summary, dATP provides a dual benefit of positive inotropy and lusitropy in cultured rat cardiomyocytes, with little alteration of Ca^2+^ transient properties, and also increases isometric force production at physiologically relevant [Ca^2+^]. These results warrant progression to animal studies to determine its potential to improve global cardiac function in normal and diseased hearts.

## Supplementary Material

01

## Figures and Tables

**Table 1:** 

	N	n	SL (μm)	Cell length(μm)
Non-transducer	5	51	1.88 ± 0.03	90.4 ± 1.8
Control(GFP)	5	50	1.84 ± 0.03	89.1 ± 1.5
R1R2(GFP)	5	52	1.82 ± 0.02	91.6 ± 1.7

**Table 2:** Contractile and Ca^2+^ transient values at 0.5 Hz stimulation.

	Fractional Shortening(%)	Maximal Shortening Rate (μm/s)	Time to Peak(ms)	Maximal Relaxation Rate (μm/s)	RT_50_ (ms)	RT_90_ (ms)	Minimal Ca^2+^(Fura ratio units)	Maximal Ca^2+^(Fura ratio units)	DT_50_ (ms)	DT_90_ (ms)
Non-transducer	6.2 ± 0.4	61.1 ± 4.4	173 ± 12	46.8 ± 6.5	208 ± 28	330 ± 59	1.10 ± 0.02	1.22 ± 0.04	246 ± 26	666 ± 74
Control(GFP)	5.5 ± 0.5	56.5 ± 4.4	217 ± 15*	37.5 ± 4.3	202 ± 25	518 ± 42*	1.12 ± 0.03	1.25 ± 0.04	297 ± 24	893 ± 63*
R1R2(GFP)	8.9 ± 0.5*	109.5 ± 8.7*	177 ± 7	117.9 ± 13.1*	113 ± 7*†	265 ± 23†	1.14 ± 0.02	1.23 ± 0.03	153 ± 10*†	435 ± 34*†

* p<0.05 as compared to No Treat,

† p<0.05 as compared to GFP,

‡ p<0.05 as compared to 0.5 Hz for all groups.

## Highlights

- dATP enhances myocardial contraction via increased crossbridge binding and kinetics
- [dATP] can be increased >10-fold via rib nucleotide reeducates (Rrm) overxpression
- Rrm over expression increased intact cardiomyocyte contractility and relaxation
- Rrm over expression had no effect on Ca^2+^ transient magnitude
- Myofilament targeted gene manipulations can improve cardiac function

## Non-standard Abbreviations and Acronyms

Rrm1: muscle ribonucleotide reductase 1
Rrm2: muscle ribonucleotide reductase 2
ARC: adult rat cardiomyocyte
GFP: green fluorescent protein
RT_50_, RT_90_: time to 50% and 90% relaxation
DT_50_, DT_90_: time to 50% and 90% Ca^2+^ decay
