# Supplementary material for: Upregulation of Cardiomyocyte Ribonucleotide Reductase Increases Intracellular 2 deoxy-ATP, Contractility, and Relaxation
Source: J Mol Cell Cardiol. Author manuscript; Available in PMC 2012 Dec 1. (PMC3208740; doi:10.1016/j.yjmcc.2011.08.026)
Supplement: 01 [file NIHMS323945-supplement-01.pdf]

METHODS AND DATA SUPPLEMENT

Plasmid design and virus production. The AdEasy™ system was used as originally described[1] to generate recombinant adenoviral vectors to express either Rrm1 or Rrm2 from the cytomegalovirus (CMV) promotor. The genes were first cloned into the shuttle vector pAdTrack-CMV. Green fluorescent protein (GFP) was also cloned in as a reporter protein to identify transduced cells via fluorescence microscopy, and a GFP-only vector was produced as a viral control. The resultant plasmid was linearized by digesting with restriction endonuclease PmeI, and subsequently co-transformed into E. coli. BJ5183 cells with an adenoviral backbone plasmid pAdEasy-1. Recombinants were selected for kanamycin resistance, and recombination confirmed by restriction endonuclease analysis. Linearized recombinant plasmid was transduced into adenovirus packaging 293 cell lines to produce high titer adenoviral preparations

Adult and Neonatal Cell Isolation and Culture. These studies were approved by the University of Washington (UW) Animal Care Committee and were conducted in accordance with federal guidelines. Animals were housed in the Department of Comparative Medicine at the UW and were cared for in accordance with the US NIH Policy on Humane Care and Use of Laboratory Animals. Neonatal Rat Cardiomyocytes (NRCs) were isolated by enzymatic dispersion from 1-3-day old newborn Fischer 344 rats as previously described[2]. Briefly, neonatal rats were decapitated and their hearts rapidly removed and placed into an ice-cold buffer (in mmol/L: NaCl 116.4, HEPES 20, NaH_2PO_4 1, glucose 5.5, KCl 5.4, $MgSO_4$ 0.8; pH 7.4). The ventricles were trimmed of atria and large vessels and cut in 2 to 3 mm pieces for repeated incubation (5-6 times @ 37°C for 25 minutes) in buffer containing collagenase type II (95 U/mL; Worthington) and pancreatin (0.6 mg/mL; Gibco BRL). After each incubation the supernatant was collected and centrifuged (600g, 5 minutes), then the resulting cell pellet was resuspended in DMEM/M199 (4:1) supplemented with 10% horse serum (ICN Flow), 5% fetal bovine serum

(HyClone), penicillin G (100 U/mL) and streptomycin (100 µg/mL) (Gibco) and preplated for 30 minutes to reduce contaminating nonmyocytes. Cardiomyocytes were pooled and counted with a typical cell yield of ≈1x10^6 per neonatal heart. The cells were then plated in media onto sterile, gel-coated 6-well dishes at a concentration of 2x10^5 cells per well for culturing and infection. After 2-4 hours, plating media was removed and cells transducted with plating media containing adenovirus (~250 viral particles per cardiomyocyte) for Rrm1 + GFP and Rrm2 + GFP or GFP-only, or left untreated. This viral titer achieved near maximal (>95%) efficiency of gene transfer with no discernable effect on cell viability (**Supplemental Figure 1**).

Adult Rat Cardiomyocytes (ARCs) were isolated by enzymatic dispersion from 6-8 week old female Fischer 344 rats similar to as previously described[3]. Rats were anesthetized with intraperitoneal injection of sodium nembutal (100mg/kg) then heparin (1mg/kg) was injected intravenously and allowed to circulate for 1 minute. The hearts were then rapidly excised and rinsed in warm (37°C) DE buffer (in mmol/L: NaCl 116.4, Pyruvate 5, HEPES 20, NaH2PO4 1, glucose 5.5, MgCl2 0.8, EGTA 100; pH 7.4) then cannulated through the aorta and perfused with digestion buffer until cleared of blood. The heart was then perfused with digestion buffer (DE buffer plus colleganase type II (120 U/mL; Worthington)) for 20-30 minutes. The ventricles were then dissected and minced and placed in fresh digestion buffer and gently agitated, then cardiomyocytes were allowed to settle for 15 minutes. The supernatant was then removed and discarded and the digestion reaction was quenched by washing the cells in DE buffer plus 2.5% bovine serum albumin (BSA; Sigma, St. Louis, MO). Cells were again allowed to settle by gravity, the supernatant removed and discarded, and cells were reintroduced to calcium by resuspending in DE buffer plus 5% BSA and 1.25 mM CaCl2. After gravity pelleting, the supernatant was removed and discarded, and the cells resuspended in DE buffer plus 5% BSA and 2.2 mM CaCl2. Finally, the cells were again gravity pelleted, the supernatant removed and discarded, and the cells are resuspended in plating media (DMEM/M199 (4:1) supplemented

with 10% horse serum (ICN Flow), 5% fetal bovine serum (HyClone), penicillin G (100 U/mL) and streptomycin (100 µg/mL) (Gibco)). The cells were then plated on to 25mm^2 glass coverslips (1.0 thickness) that were pre-coated with laminin (in PBS). After 2-4 hours, plating media was removed and cells transduced with plating media containing adenovirus (~250 viral particles per cardiomyocyte) overnight. Cardiomyocytes were then maintained and fed daily with plain DMEM.

Contractile Assessments. Cell shortening and relengthening was monitored and recorded using IonOptix SarcLen system video microscopy. (IonOptix, Milton, MA, USA). For all cells, video microscopy was completed using a 40x objective (Olympus UWD 40) and 25x intermediate lenses. Contractile assessments were performed at room temperature (22-24°C) and in fresh buffer (in mmol/L: $CaCl_2$ 1.8, $MgCl_2$ 1.0, KCl 5.4, NaCl 140, HEPES 10, NaH_2PO_4 0.33, glucose 5,; pH 7.4). For adult cardiomyocytes, only cells that followed field stimulation 1:1 and with resting sarcomere lengths above 1.70 µm were measured. Average cardiomyocyte length and sarcomere length is noted in **Table 1** of the manuscript with high and low values for length of 75.4 µm and 134.1 µm for non-transduced, 74.0 µm and 121.2 µm for GFP transduced, and 72.4 µm and 132.9 um for Rrm1+Rrm2 transduced. Experiments were performed by 3 different experimentalists, with at least 2 rotating on a given day, and all data has been analyzed in duplicate by 3 different analysts. No differences were found between experimentalists or analysts. Single cardiomyocytes in the cell bath were field-stimulated with a 4 ms square supra-threshold (10V) pulse at 0.5, 1 and 2 Hz through parallel platinum electrodes. Cell shortening was recorded by illuminating the myocytes with red transmitted light (> 600 nm). Calcium transients induced by electrical stimulation were measured in Fura2 loaded adult cardiomyocytes using IonOptix equipment as described[4]. Briefly, calcium transients were recorded by measuring Fura-2 fluorescence passed through a 510 nm emission filter to a photomultiplier tube using the interpolated pseudo-ratiometric method with 380 nm excitation

during, and 360 nm excitation at the onset and end of, 20 second recording events. Fura2 fluorescence was measured using an IonOptix spectrophotometer (Stepper Switch) attached to a fluorescence microscope. Emitted Fura2 fluorescence was collected by the 40X objective, passed through a 510nm filter and detected by a photomultiplier tube. The cell and sarcomere length, and fluorescent signals, were recorded simultaneously by computer acquisition, and were analyzed later using proprietary software (IonOptix). Contractile measurements of adult cardiomyocytes taken at 1 Hz and 2 Hz stimulation frequency are summarized in **Supplemental Table 1** and **Supplemental Table 2**, respectively. There was functional potentiation (increased rate and extent of shortening and rate of relaxation) at all frequencies in Rrm1 + Rrm2 transduced cardiomyocytes. Response to increased stimulation frequency was similar between groups and primarily involved a shortening in the times to relaxation, but GFP-only transduced cardiomyocytes experienced elevations in minimal and maximal Ca^{2+} with increasing frequency, which did not occur in Rrm1 + Rrm2 transduced cardiomyocytes. Finally, a subset of experiments was performed at 37°C (**Supplemental Table 3**) and demonstrate that the rate and extent of shortening was increased in all groups as compared to 22-24°C, but that contractile potentiation is maintained in Rrm1+Rrm2 transduced myocytes over non-transduced and GFP transduced myocytes. Similarly, the rates of Ca^{2+} release and re-uptake were also increased at 37°C vs. room temperature, with Rrm1+Rrm2 overexpression resulted in faster Ca^{2+} transient decay as was observed at ambient temperature.

For neonatal rat cardiomyocytes, spontaneously beating cardiomyocytes were arbitrarily selected. Due to typical neonatal cardiomyocyte asymmetry, care was taken to align contraction along the major axis of myocyte movement, and all measurements were normalized to 'resting' length. Experiments showed that neonatal cardiomyocytes maintain beating rate and amplitude for over an hour under the conditions noted above. There was no difference in the intrinsic beating frequency between non-transduced cardiomyocytes and those transduced with

GFP-only or Rrm1+Rrm2 + GFP. This implies that exposure to adenovirus and/or overexpression of GFP or Rrm1+Rrm2 did not alter the intrinsic cardiomyocyte pacemaker potential. Contractility of neonatal cardiomyocytes transduced with Rrm1+Rrm2 was greatly increased, more than doubling the rate and extent of shortening, similar to increased contractility seen in adult cardiomyocytes. Contractility of GFP-only transduced cardiomyocytes was compromised, as compared to non-transduced myocytes, with a slower rate and decreased extent of shortening. However, relaxation, as indicated by early and late phase cell relengthening (10%, 50%, and 90% relaxation times (RT)), was not impaired. The initial maximal rate of relaxation was increased by Rrm1+Rrm2, but reflected the extent and rate of shortening amongst groups (**Table 1**). Both early and late phase cell relengthening (indicated from 10%, 50%, and 90% relaxation times (RT) did not differ between GFP and Rrm1+Rrm2 transduced neonatal cardiomyocytes. Example contractile transients of neonatal cardiomyocytes are shown in **Supplemental Figure 2**, and measurements are summarized in **Supplemental Table 4**.

Statistical Analysis. Maximal cardiomyocyte shortening and relengthening and calcium transient rise and decay were calculated offline using IonOptix software to determine the maximum of the first derivative of these transients. Times to peak shortening and 50% and 90% return to baseline were also calculated offline. To convert the fluorescence data to $[Ca^{2+}]_i$ an *in vitro* calibration of the measured ratio (R) was performed using the Grynkiewicz equation[5]:

$$[Ca] = Kd * (R-Rmin) / (Rmax - R) * Sf2 / Sb2.$$

The values of Rmax and Rmin are the ratio values measured under conditions of saturating calcium levels and in the absence of calcium, respectively. The values of Sb2 and Sf2 are proportional to the fluorescence excited by the denominator wavelength (380nm) again under conditions of saturating (b, bound) calcium levels and in the absence (f, free) of calcium,

respectively. A value of 225 nmol/L was assumed for the dissociation constant for the fura2-calcium binding (Kd). Values for minimal and maximal $[Ca^{2+}]_i$ as determined from this equation or shown in **Supplemental Table 5**, and differences between groups are statistically identical to those determined from the ratiometric values. Furthermore, these $[Ca^{2+}]_i$ values are in agreement with those previously reported by Herron et al., using similar conditions (i.e., adenovirally transduced adult rat cardiomyocytes after 2 days in culture)[6]. Data reflect the averages of individual cardiomyocytes taken from 5 hearts per group. All observed differences were maintained even when averaged on a heart-by-heart basis, as compared to the cell-by-cell basis as reports. This is demonstrated in **Supplemental Table 6**, where "by heart" averages are compared to "by cell" averages as reported in **Table 2** of the main manuscript. Statistical differences were determined by ANOVA, with Student-Newman-Keuls as a *post-hoc* pairwise test (SigmaPlot 11). Trabeculae were compared using paired t-tests. Differences at the p-value < 0.05 were considered statistically significant. Values are shown as mean ± S.E.M., unless indicated otherwise.

RT-PCR and Western blotting. RT-PCR and western blot analysis of RRM transcript and protein abundance, respectively, is shown in **Supplemental Figure 3**. Neonatal cardiomyocytes (~1-2x10^5) were harvested from individual wells from a 6-well tissue culture dish by mild (0.05%) trypsin treatment. For determination of total cellular Rrm content, intact myocytes were immediately stored in Laemmli sample buffer at -80 °C until SDS-PAGE. SDS-PAGE separated proteins were transferred to nitrocellulose membrane and probed with monoclonal antibodies specific to Rrm1 or Rrm2 (Santa Cruz Biotechnology, Santa Cruz, CA), after blocking with 5% milk (w/v in Tris-buffered saline), which showed significant overexpression of Rrm1 and Rrm2 in transduced cells. Protein bands were quantified using open access software (ImageJ, NIH) and expression of each Rrm isoform was expressed relative to the housekeeping protein GAPDH. Rrm1 overexpression in neonatal rat cardiomyocytes led to significantly increased Rrm1 mRNA (**Supplemental Figure 3A**), with GAPDH as a loading control. Similarly, Rrm2

overexpression in neonatal cardiomyocytes also had significantly increased respective transcript abundance (**Supplemental Figure 3B**) when normalized to GAPDH.

Transient kinetics and data analysis. Rapid kinetic measurements were taken at 10 °C (**Supplemental Figure 4**) and 20 °C (**Supplemental Figure 5**) with a standard Hi-Tech Scientific SF-61 DX2 stopped-flow system fitted with ceramic valves and a 75 W Xe–Hg lamp and monochromator for wavelength selection. Pyrene fluorescence on actin was excited at 365 nm and emission monitored through a KV 398 nm cutoff filter as described previously[7]. The stopped-flow transients were fitted to one or two exponentials by non-linear least squares curve fitting using the Kinetic Studio software (TgK Scientific). All experiments were carried out in 20 mM Cacodylate buffer, pH 7.0 containing 100 mM KCl, and 5 mM $MgCl_2$. Rabbit skeletal muscle actin was purified as previously described[8]. Skeletal muscle myosin S1 was prepared from rabbit bulk muscle (fast), rabbit soleus and mouse heart based on Margossian and Lowey[9] and Weeds and Taylor[10] with some modifications[7].

The results of the kinetic interaction of actin –S1 with nucleotide were interpreted in terms of the model in **Scheme 1**, which we described previously for fast and slow skeletal muscle myosin[7]. This is based on the scheme originally proposed by Geeves, Perrault and Coluccio[11] for non-muscle myosin class I.

$$\underset{\text{CLOSED}}{A{\cdot}M'} \underset{k_{-\alpha}}{\overset{K_{\alpha}\;\; k_{+\alpha}}{\rightleftharpoons}} \underset{\text{OPEN}}{A{\cdot}M} + T \overset{K_1}{\rightleftharpoons} \underset{\text{OPEN}}{A{\cdot}M{\cdot}T} \overset{k_{+2}}{\rightleftharpoons} A{\sim}M{\cdot}T \xrightarrow{k_{dissn}} A + M{\cdot}T \underset{k_{-3}}{\overset{k_{+3}}{\rightleftharpoons}} A + M{\cdot}D{\cdot}P_i$$

In the scheme, the equilibrium constants are defined in the direction from A·M' towards M·T, thus K_1 is an association constant. A very rapid equilibrium is reached between A·M and ATP after mixing actin-S1 with ATP, defined by the association constant K_1 and followed by isomerization of the ternary complex (A·M·T), which limits the maximum rate of actin

dissociation from the complex (k_{+2}). Thus, the observed rate constant for the ATP-induced actin-S1 dissociation is defined by the equation 1:

$$k_{obs} = k_{+2}K_1 \frac{[ATP]}{1+K_1[ATP]}$$ **equation 1**

For some myosins i k(like slow skeletal myosin), an additional slow time course component is seen with a rate constant defined by $k_{+\alpha}$ and the relative amplitudes of the fast and slow phases defined by the equation 2 :

$$\frac{A_{fast}}{A_{slow}} = \frac{[A \cdot M^{open}]}{[A \cdot M'^{closed}]} \equiv K_{\alpha}$$ **equation 2**

For fast skeletal myosin, if the closed form exists, the term K_{α} >> 1 and only a single phase is observed. There were no differences observed at either temperature between ATP and dATP for a given myosin isoform.

Supplemental Table 1: Contractile and Ca^{2+} transient values at 1 Hz stimulation.

	Fractional Shortening (%)	Maximal Shortening Rate (µm/s)	Time to Peak (ms)	Maximal Relaxation Rate (µm/s)	RT_{50} (ms)	RT_{90} (ms)	Minimal Ca^{2+} (Fura ratio units)	Maximal Ca^{2+} (Fura ratio units)	DT_{50} (ms)	DT_{90} (ms)
Non-transduced	6.2 ± 0.4	61.9 ± 5.7	189 ± 15	41.0 ± 5.5	191 ± 21	317 ± 27	1.07 ± 0.03	1.24 ± 0.04	191 ± 13	478 ± 23
Control (GFP)	6.9 ± 0.6	63.2 ± 6.4	184 ± 8	39.7 ± 4.7	156 ± 15	365 ± 25	1.23 ± 0.03	1.39 ± 0.04	206 ± 11	511 ± 27
R1R2 (GFP)	10.7 ± 0.9*†	126.8 ± 9.2*†	178 ± 13	150.2 ± 4.2*†	110 ± 12*	209 ± 22*†	1.14 ± 0.03	1.27 ± 0.03	103 ± 9*†	262 ± 20*†

* = p<0.05 as compared to Non-Transduced, † = p<0.05 as compared to GFP, ‡ = p<0.05 as compared to 0.5 Hz for all groups.

Supplemental Table 2: Contractile and Ca^{2+} transient values at 2 Hz stimulation.

	Fractional Shortening (%)	Maximal Shortening Rate (μm/s)	Time to Peak (ms)	Maximal Relaxation Rate (μm/s)	RT_{50} (ms)	RT_{90} (ms)	Minimal Ca^{2+} (Fura ratio units)	Maximal Ca^{2+} (Fura ratio units)	DT_{50} (ms)	DT_{90} (ms)
Non-transduced	6.9 ± 0.5	85.3 ± 6.7	155 ± 6	61.6 ± 7.0	92 ± 5	174 ± 7	1.09 ± 0.02	1.24 ± 0.04	117 ± 6	237 ± 10
Control (GFP)	5.7 ± 1.0	55.3 ± 8.9	157 ± 8	34.3 ± 6.1	113 ± 10	251 ± 43	1.28 ± 0.06	1.45 ± 0.08	141 ± 10	277 ± 27
R1R2 (GFP)	10.1 ± 0.8*†	131.2 ± 12.3*†	163 ± 10	98.3 ± 7.7*†	96 ± 8	166 ± 11	1.17 ± 0.04	1.26 ± 0.04	96 ± 7*	182 ± 11*

* = p<0.05 as compared to Non-Transduced, † = p<0.05 as compared to GFP, ‡ = p<0.05 as compared to 0.5 Hz for all groups.

Supplemental Table 3: Contractile and Ca^{2+} transient values at 37°C at 0.5 Hz stimulation.

	Fractional Shortening (%)	Maximal Shortening Rate (μm/s)	Time to Peak (ms)	Maximal Relaxation Rate (μm/s)	RT_{50} (ms)	RT_{90} (ms)	Minimal Ca^{2+} (Fura ratio units)	Maximal Ca^{2+} (Fura ratio units)	DT_{50} (ms)	DT_{90} (ms)	Response (FS/Max Ca^{2+})
Control (GFP)	6.5 ± 0.9	152.7 ± 9.5	109 ± 6	134.1 ± 24.7	57 ± 8	176 ± 32	1.07 ± 0.02	1.16 ± 0.02	139 ± 27	275 ± 37	6.8 ± 0.8
R1R2 (GFP)	9.2 ± 0.7*	189.0 ± 9.6*	113 ± 7	156.8 ± 28.3	58 ± 10	176 ± 63	1.07 ± 0.02	1.17 ± 0.03	93 ± 15*	266 ± 33	9.3 ± 0.8

* = p<0.05 as compared to Non-Transduced

Supplemental Table 4: Contractile measurements of neonatal rat cardiomyocytes.

	N	Beat Rate	Fractional Shortening (%)	Maximal Shortening Rate (CL/s)	Maximal Relaxation Rate (CL/s)	RT_{10} (ms)	RT_{50} (ms)	RT_{90} (ms)
Non-transduced	26	71 ± 3	4.7 ± 0.5	3.1 ± 0.5	2.3 ± 0.2	60 ± 1	156 ± 21	271 ± 45
Control (GFP)	20	71 ± 4	4.2 ± 0.5	2.7 ± 0.3	2.3 ± 0.3	57 ± 8	153 ± 24	237 ± 41
RRM1/2 + GFP	35	70 ± 3	10.2 ± 0.8*†	9.2 ± 1.2*†	7.0 ± 1.0*†	65 ± 8	142 ± 15	241 ± 23

CL/s = Cell lengths per second; RT_{10}, RT_{50}, RT_{90}, times to 10%, 50%, and 90% relaxation, respectively. * Significant difference from non-transduced, † Significant difference from Control (GFP) (p<0.05).

Supplemental Table 5: Minimal and maximal [Ca2+] as calculated by the Grynkiewicz equation.

	Minimal Ca^{2+} @ 0.5 Hz (nmol/L)	Maximal Ca^{2+} @ 0.5 Hz (nmol/L)	Minimal Ca^{2+} @ 1 Hz (nmol/L)	Maximal Ca^{2+} @ 1 Hz (nmol/L)	Minimal Ca^{2+} @ 2 Hz (nmol/L)	Maximal Ca^{2+} @ 2 Hz (nmol/L)
Non-transduced	146 ± 17	324 ± 14	132 ± 14	327 ± 24	130 ± 18	307 ± 17
Control (GFP)	157 ± 13	305 ± 17	228 ± 14*	445 ± 21*	287 ± 18*	543 ± 16*
R1R2 (GFP)	157 ± 16	305 ± 7	201 ± 13	307 ± 12	190 ± 21	309 ± 22

* = p<0.05 as compared to Non-Transduced

Supplemental Table 6: Cell-by-cell vs. heart-by-heart analysis at 0.5 Hz stimulation.

	Fractional Shortening (%)	Maximal Shortening Rate (µm/s)	Maximal Relaxation Rate (µm/s)	RT_{50} (ms)	RT_{90} (ms)	Minimal Ca^{2+} (Fura ratio units)	Maximal Ca^{2+} (Fura ratio units)	DT_{50} (ms)	DT_{90} (ms)
Non-transduced	6.2 ± 0.4	61.1 ± 4.4	46.8 ± 6.5	208 ± 28	330 ± 59	1.10 ± 0.02	1.22 ± 0.04	246 ± 26	666 ± 74
By Heart	6.5 ± 0.3	64.6 ± 6.7	48.4 ± 6.5	204 ± 34	362 ± 67	1.10 ± 0.08	1.20 ± 0.05	261 ± 29	696 ± 23
Control (GFP)	5.5 ± 0.5	56.5 ± 4.4	37.5 ± 4.3	202 ± 25	518 ± 42*	1.12 ± 0.03	1.25 ± 0.04	297 ± 24	893 ± 63*
By Heart	5.1 ± 1.2	56.7 ± 2.4	37.1 ± 10.7	205 ± 12	527 ± 17*	1.14 ± 0.08	1.26 ± 0.07	285 ± 42	843 ± 87*
R1R2 (GFP)	8.9 ± 0.5*	109.5 ± 8.7*	117.9 ± 13.1*	113 ± 7*†	265 ± 23†	1.14 ± 0.02	1.23 ± 0.03	153 ± 10*†	435 ± 34*†
By Heart	9.1 ± 0.9*	111.5 ± 3.3*	118 ± 10.5*	117 ± 23*†	271 ± 26*†	1.15 ± 0.07	1.26 ± 0.08	156 ± 18*†	419 ± 47*†

* = p<0.05 as compared to Non-Transduced, † = p<0.05 as compared to GFP

FIGURE LEGENDS

Supplemental Figure 1: Cultured neonatal rat cardiomyocytes following adenoviral transduction with Rrm1 + GFP and Rrm2 + GFP under white light (A) and fluorescent microscopy (B). 20X magnification

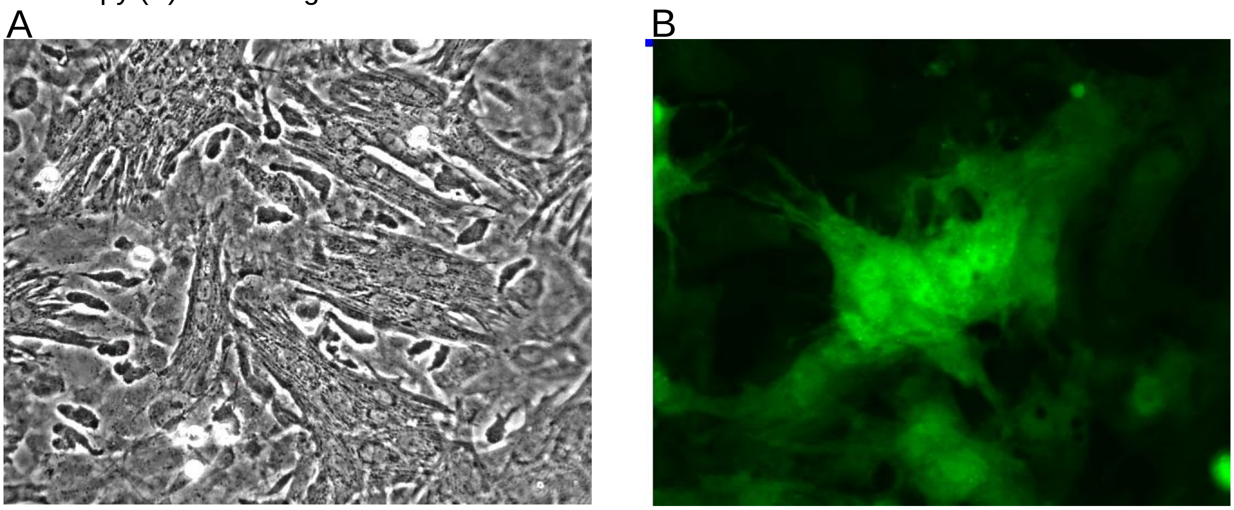

Figure 2: (A) Representative neonatal cardiomyocyte shortening traces from non-transduced (black), GFP-only (green), and Rrm1+Rrm2 (blue) transduced cardiomyocytes. Rrm1+Rrm2 transduced cardiomyocytes had a significantly increased extent and rate of shortening (B), while the intrinsic beating frequency and time to relaxation were unaffected.

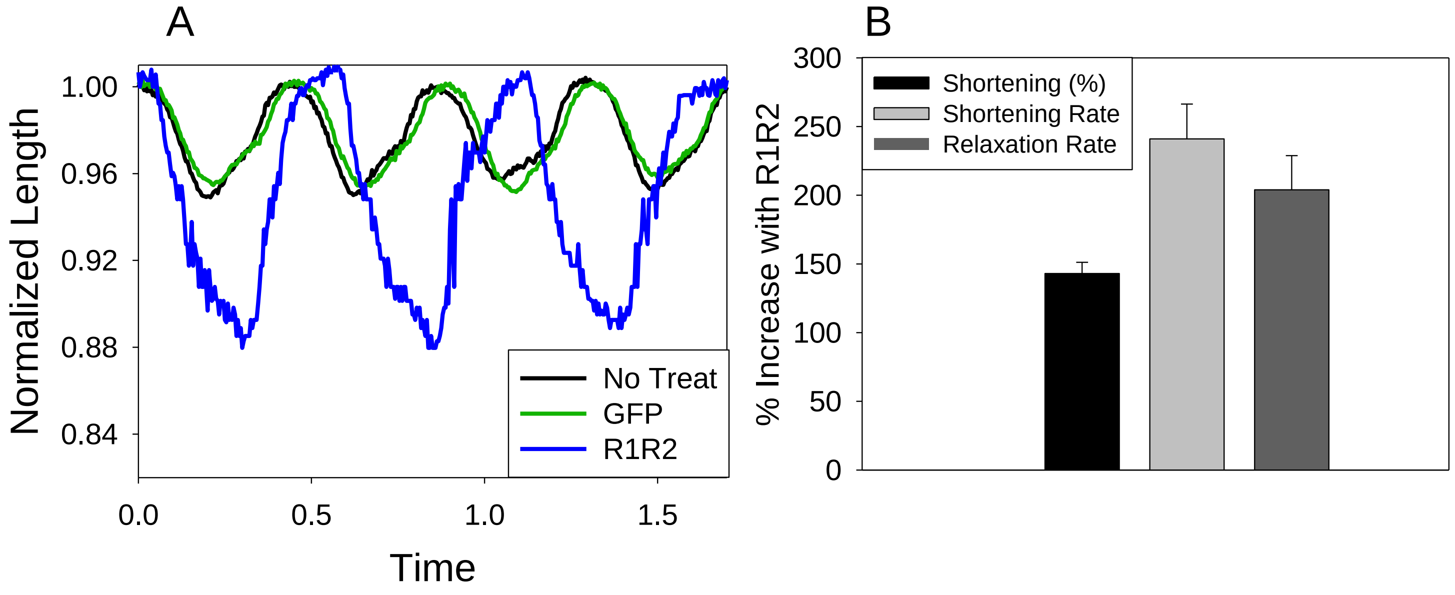

Figure 3: (A) RT-PCR analysis of transduced neonatal cardiomyocytes demonstrated increased Rrm1 mRNA in transduced cells compared to control (non-transduced) cells. (B) RT-PCR analysis of transduced neonatal rat cardiomyocytes demonstrated increased Rrm2 mRNA in transduced cells compared to control (non-transduced) cells.

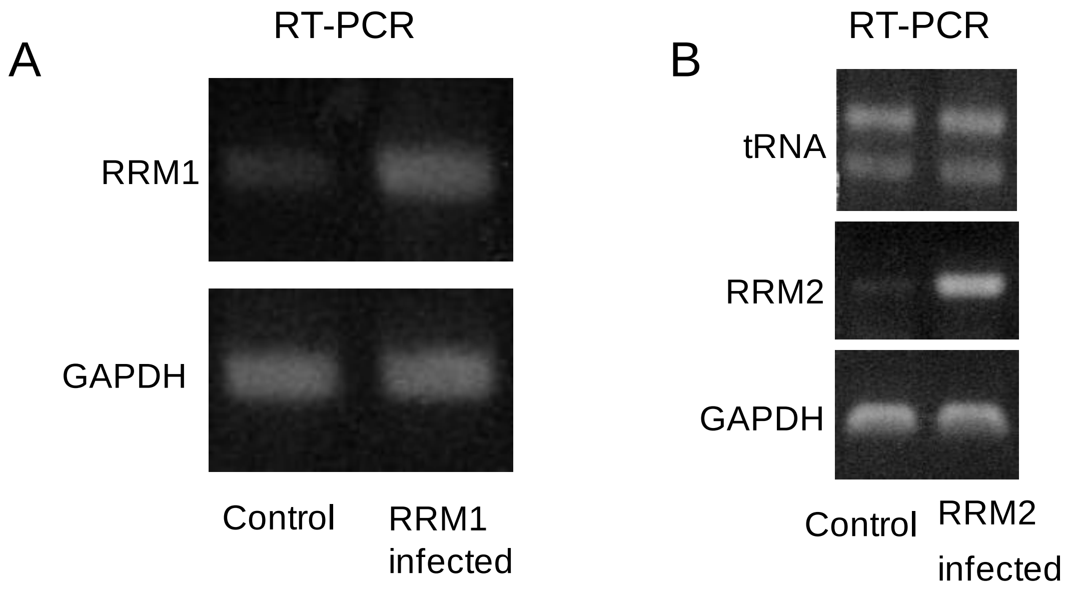

Figure 4: Rapid kinetic measurements of nucleotide binding and actin-myosin dissociation taken at 10°C (A) and 20°C (B). There was no difference in k_{obs} between ATP and dATP at any [NTP] at either temperature.

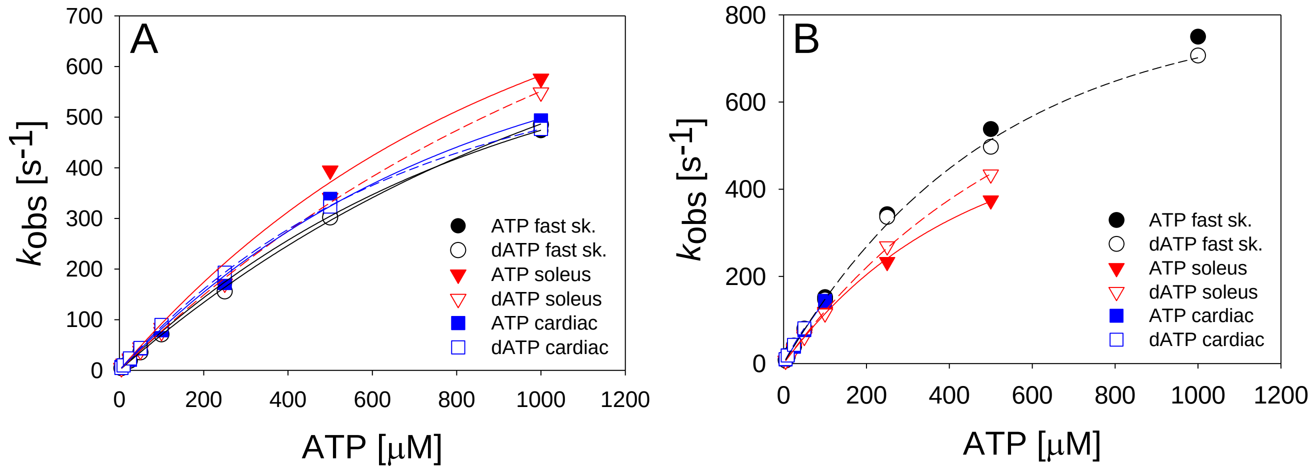

1. He TC, Zhou S, Da Costa LT, Yu J, Kinzler KW, Vogelstein B. A simplified system for generating recombinant adenoviruses. *Proc Natl Acad Sci U S A.* 1998;95:2509-2514.

2. Reinecke H, Zhang M, Bartosek T, Murry CE. Survival, integration, and differentiation of cardiomyocyte grafts: a study in normal and injured rat hearts. *Circulation.* 1999;100:193-202.

3. Santana LF, Kranias EG, Lederer WJ. Calcium sparks and excitation-contraction coupling in phospholamban-deficient mouse ventricular myocytes. *J Physiol.* 1997;503(1):21-29.

4. Herron TJ, Devaney E, Mundada L, Arden E, Day S, Guerrero-Serna G, Turner I, Westfall MV, Metzger JM. Ca2+-independent positive molecular inotropy for failing rabbit and human cardiac muscle by alpha-myosin motor gene transfer. *Faseb J.* 2010;24(2):415-424.

5. Grynkiewicz G, Poenie M, Tsien RY. A new generation of Ca2+ indicators with greatly improved fluorescence properties. *J Biol Chem.* 1985;6:3440-3450.

6. Herron TJ, Vandenboom R, Fomicheva E, Mundada L, Edwards T, Metzger JM. Calcium-independent negative inotropy by beta-myosin heavy chain gene transfer in cardiac myocytes. *Circ Res.* 2007;100(8):1182-1190.

7. Iorga B, Adamek N, Geeves MA. The slow skeletal muscle isoform of myosin shows kinetic features common to smooth and non-muscle myosins. *J. Biol. Chem.* 2007;982(6):3559-3570.

8. Spudich JA, Watt S. The regulation of rabbit skeletal muscle contraction. I. Bioechemical studies of the interaction of the tropomyosin-troponin complex with actin and the proteolytic fragments of myosin. *J. Biol. Chem.* 1971;246(15):4866-4871.

9. Margossian SS, Lowey S. Preparation of myosin and its subfragments from rabbit skeletal muscle. *Methods Enzymol.* 1982;85(B):55-71.

10. Weeds AG, Taylor RS. Separation of subfragment-1 isoenzymes from rabbit skeletal muscle myosin. *Nature.* 1975;257(5521):54-56.

11. Geeves MA, Perreault-Micale C, Coluccio LM. Kinetics analyses of a truncated mammalian myosin I suggest a novel isomerization event preceding nucleotide binding. *J. Biol. Chem.* 2000;275(28):21624-21630.
